# Freshwater bryozoans of Lithuania (Bryozoa)

**DOI:** 10.3897/zookeys.774.21769

**Published:** 2018-07-12

**Authors:** Ingrida Šatkauskienė, Timothy Wood, Jurgita Rutkauskaitė-Sucilienė, Vida Mildažienė, Simona Tučkutė

**Affiliations:** 1 Vytautas Magnus University, Faculty of Natural Sciences, Department of Biology, Vileikos str. 8-802, 44404 Kaunas, Lithuania; 2 Wright State University, Department of Biological Sciences, Dayton, OH 45435, USA; 3 Lithuanian Energy Institute, 3 Breslaujos str., LT-44403, Kaunas, Lithuania

**Keywords:** Phylactolaemata, *Plumatella*, statoblasts, bryozoa, Lithuania

## Abstract

Nine species of freshwater bryozoans were recorded in Lithuania in a survey of 18 various types of freshwater bodies. Eight species were assigned to the Class Phylactolaemata and families Plumatellidae and Cristatellidae (*Plumatella repens*, *Plumatella fungosa*, *Plumatella fruticosa*, *Plumatella casmiana*, *Plumatella emarginata*, *Plumatella geimermassardi*, *Hyalinella punctata* and *Cristatella mucedo*). The ninth species, *Paludicella articulata*, represented the Class Gymnolaemata.

*Plumatella geimermassardi* and *P. casmiana* were recorded for the first time in Lithuania. For the plumatellids, species identification was achieved partly by analysing statoblasts’ morphological ultrastructures by scanning electron microscopy.

## Introduction

Freshwater bryozoans grow in colonies of minute tentacle-bearing clones (zooids) that feed upon microscopic plankton. They are often found in ponds, lakes, and rivers, forming a cryptic but often a significant part of the aquatic fauna (Bushnell 1966). Bryozoans are important to ecosystems as filter feeders (Wood et al. 2006), extracting phytoplankton from the water and producing faecal pellets that nourish benthic meiofauna (Bushnell and Rao 1974). The colony structure also creates important habitat and shelter for other organisms: protozoans, rotifers, ostracods, nematodes and chironomids (Ricciardi and Reiswig 1994). Bryozoans hosting certain myxozoan parasites can spread proliferative kidney disease in fish, which is often fatal in farmed and wild fish populations (Grabner and ElMatbouli 2008; Bartošová-Sojková et al. 2014).

In their natural habitat freshwater bryozoans are easily overlooked and, in many areas, there is little information on the identity or distribution of species.

Fundamental studies of freshwater bryozoans in Europe were launched with a pioneering monograph by Allman (1856), which established the Class Phylactolaemata as an exclusively freshwater group and named Plumatellidae as the largest family within that class. A second monograph by Jullien (1885) described species from France following Allman’s taxonomy. Shortly afterwards a third monograph appeared from Germany (Kraepelin 1887) proposing a new taxonomic scheme for the plumatellids which has by now fallen out of use. Since those early years there have been bryozoan surveys from a number of European countries, including the Netherlands (Lacourt 1949), Sweden (Borg 1941), Italy (Viganó 1965, Luxembourg (Geimer and Massard 1986), Belgium (Loppens 1906), Bulgaria (Gruncharova 1968), Ireland (Smyth 1994), and Britain (Mundy 1980). In most of these works only 5–8 species were documented.

Until recently freshwater bryozoans in the Baltic region were known only from a brief paper from Latvia (Trauberg 1940). Six species were listed using the Kraepelin taxonomic scheme. They included Plumatella polymorpha Krpln. var. repens (L.), Krpln., Plumatella polymorpha Krpln. var. appressa Krpln., Plumatella polymorpha Krpln. var. fungosa (Pall.) Krpln., Plumatella princeps Krpln. var. emarginata Allm., *Cristatella mucedo* Cuv., and *Paludicella ehrenbergi* Bened. Trauberg (1940) provided measurements of certain colonies and statoblasts and included some ecological information as well.

In 2015 an old master’s thesis was uncovered in Lithuania with a detailed account of bryozoans from the area (Satkauskiene et al. 2018). Written by Bronė Pajiedaitė (1933) the work covered a period of 1931–1933. Pajiedaitė collected bryozoans from widely scattered locations in Lithuania (five districts and approximately eleven localities) including lakes, ponds, and rivers. From microscopic examinations, she illustrated colonies and statoblasts, prepared notes on associations with other organisms, and described substrata on which common bryozoans were found. During her studies, Bronė Pajiedaitė prepared at least 12 bottles of fixed whole specimens and 70 high quality microscope slides (Satkauskiene et al. 2018). Unfortunately, the whole specimens were apparently destroyed during World War II (1941–1945), but the surviving microscopic slides are now deposited in Vilnius University. Altogether Pajiedaitė described seven species of freshwater bryozoans in detail: *Paludicella articulata* (Ehrenberg, 1831), *Cristatella mucedo* (Cuvier, 1798), *Plumatella fungosa* (Pallas, 1768), *Plumatella repens* (Linnaeus, 1758), *Plumatella emarginata* (Allman, 1844), *Plumatella fruticosa* (Allman, 1844), and *Hyalinella punctata* (Hancock, 1850).

In recent years the number of freshwater bryozoans documented from Europe has grown to 19 (Massard and Geimer 2005, 2008a; Wood and Okamura 2004). Meanwhile from seven to eleven freshwater bryozoan species have been reported in countries neighbouring Lithuania: Latvia, Poland, and Belarus (Kaminski 1984, Fauna Europea 2013). Based on the species diversity in other European countries, we can expect a more diverse list of bryozoans in Lithuania as well.

The present work describes freshwater bryozoans studied in 18 freshwater bodies in Lithuania.

## Materials and methods

### Climate of Lithuania

Lithuania is distinguished by a highly diverse geography: plains, hills, abundant forests, lakes, wetlands, and Baltic Sea. The climate of the Lithuania can be described as typical European with strong continental influence providing warm summers and fairly severe winters. The weather is often windy and humid due to the proximity of the Baltic Sea.

The average air temperature is 7.2 °C. July is the warmest month with an average temperature of 18 °C. January and February are the coldest months with average temperatures around -3.35 °C, but sometimes winter days can be much colder with temperatures about -32.4 °C. Annual precipitation ranges from 560 to 700 mm. Snow cover can last from 60 to 90 days. The flat landscape retains much of the precipitation, which leads to a relatively high water level (Lithuanian Hydrometeorological Service under the Ministry of Environment).

### Characteristic of sampling sites

**Figure 1.**
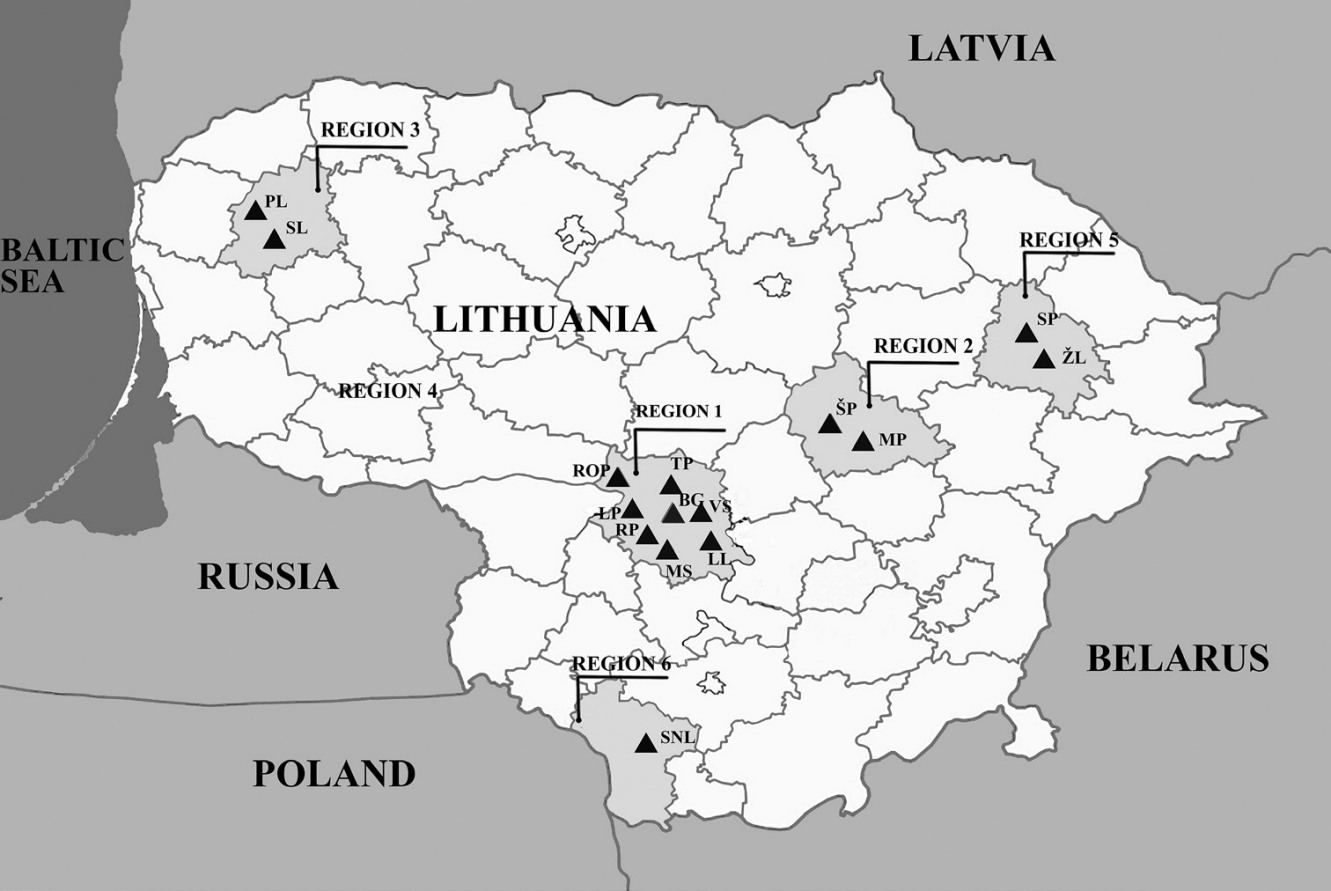
Map of the investigated regions in Lithuania. Triangles mark the approximate locations of collecting sites, which are further identified by name abbreviations (see Table 1).

Our bryozoan survey was conducted during April through October 2015–2017. We investigated localities that included different types of water bodies: lakes, ponds, lagoons and lotic habitats (streams and rivers). Fig. 1 shows regions in Lithuania that were surveyed. Geographical details and descriptions of collecting sites are listed below and summarized in Table 1.

**Table 1.** Summary of collecting sites, their locations, abbreviation of locations names, and the bryozoan species collected. Bryozoan species are expressed by the first letter of the genus followed by the first three letters of the species.

	District	Site and name abbreviation	Coordinates	Species
1	Kaunas City	Pond, Kaunas Botanical Garden, BG	54°52'20.5"N, 23°54'45.4"E	PREP; PFUN; PFRU; PGEI; CMUC
1	Kaunas	Linksmakalnis pond, LP	54°45'31.3"N, 23°55'20.5"E	PREP; PFUN; PFRU; PCAS
1	Kaunas	Raudondvaris pond, RP	54°59'10.7"N, 23°46'12.5"E	PREP
1	Kaunas	Rokai pond, ROP	54°50'01.2"N, 23°57'21.9"E	PREP; PFRU; CMUC
1	Kaunas	Tribalė pond, TP	54°50'23.3"N, 23°51'30.0"E	PREP; PFRU
1	Kaunas	Lampėdžiai lake, LL	54°54'54.3"N, 23°49'29.7"E	PREP; PCAS; PGEI; CMUC
1	Kaunas	Maišia stream, MS	54°49'47.8"N, 23°52'13.4"E	PREP; PFUN; PCAS; PGEI
1	Kaunas	Veršvio stream, VS	54°55'39.8"N, 23°52'09.6"E	PREP; HPUN
2	Ukmergė	Šventupė pond, ŠP	55°19'20.4"N, 24°53'07.4"E	PREP; PFRU; PEMA
2	Ukmergė	Mūša pond, MP	55°18'33.5"N, 24°49'38.6"E	PREP; PFRU; PCAS; PFUN
3	Plungė	Plateliai Lake, PL	56°02'41.9"N, 21°51'35.5"E	PREP; PFRU, PCAS; PGEI
3	Plungė	Skyplaičiai Lake, SL	56°00'46.2"N, 21°56'15.9"E	PREP; PFRU; PFUN; PGEI
4	Trakai	Škilietai Lake, ŠL	54°37'01.5"N, 24°30'33.5"E	PREP; PFUN; PFRU;
4	Vilnius and Trakai	Elektrėnai Reservoir, EL	54°45'34.4"N, 24°40'16.5"E	PREP; PFUN; PCAS; PGEI; CMUC
4	Trakai and Kaunas	Strėva river, SR	54°35'03.8"N, 24°41'57.4"E	PREP; PCAS; PGEI
5	Utena	Saterečius pond, SP	55°39'55.7"N, 25°42'00.3"E	PREP; PFRU; PGEI
5	Utena	Žvirgždelis Lake, ŽL	55°42'02.1"N, 25°41'34.9"E	PREP; PFRU
6	Lazdijai	Snaigynas Lake, SNL	54°05'35.4"N, 23°44'03.0"E	PREP; PFRU; CMUC; PART

### Region 1

**Pond in Kaunas botanical garden** (located in Kaunas city). Small eutrophic pond with abundant macro- and microalgae. The bottom is sludge. Water pH is 7.89.

**Linksmakalnis pond** (Kaunas district). Large artificial pond, what shorelines are overgrown by *Phragmites* sp. The bottom is sandy. Water pH is 7.18.

**Raudondvaris**, **Rokai** and **Tribalė ponds**. All these ponds located in Kaunas district and have similar characteristics: the bottom is sand mixed with sludge, the littoral is overgrown by *Acorus calamus* and *Phragmites* sp. in Raudondvaris and Tribalė ponds. Vegetation on the shores of Rokai pond are rare, water birds are common here.

Water pH varies from 7.51–7.85 (Rokai and Tribalė ponds respectively) to pH 8.35 in Raudondvaris pond.

**Lampėdžiai lake** (located in Kaunas city). Relative large (1,252 km²), semi-artificial lake. The bottom is sandy. Shorelines are without the trees, only *Phragmites* sp. occurs occasionally in the littoral. Water pH is 8.06.

**Maišia stream** (located in the outskirts of Kaunas). One side of shore is overgrown by deciduous trees, *Phragmites* sp. and *Typha angustipholia*. Water is polluted by sewage. Water pH is 7.48.

**Veršvio stream** (located in western part of Kaunas city). Small and shallow stream, that dries up in the summer. Shore is lined by trees and shrubs. Bottom is sandy. Water pH is 7.79.

### Region 2

**Šventupė pond** (Ukmergė district). The shoreline is overgrown by shrubs and other vegetation. *Phragmites* sp., *Acorus calamus*, *Lemna minor* dominates in littoral. A small stream enters in one end of pond. Another end of the pond is connected with Šventoji River. Water pH 7.40.

**Mūša pond** (Ukmergė district). Mūšia stream enters in this artificial pond. *Phragmites* sp., *Acorus calamus* and *Nymphaea lutea* occur in the littoral. Water pH is 7.81.

### Region 3

**Plateliai Lake** (Plungė district) is the large lake covering about 12 km² with a maximum depth of 47 m. Water is contributed by seventeen small streams. The Bottom is sandy in the collecting sites.

**Skyplaičiai Lake** (Plungė district) covers 0.068 km² and is surrounded by a mixed deciduous forest. The bottom is muddy; shorelines are overgrown by *Phragmites* sp. According to the EU Habitats Directive, this lake is notable for its Charophyta communities.

### Region 4

**Škilietai Lake** (Trakai district) covers about 0.033 km² with maximum depth of 12 m. The lake is surrounded by pine forest.

**Elektrėnai Reservoir** (Vilnius district and Trakai district) is the third largest artificial lake in Lithuania. The reservoir measures about 0.0126 km². The lake is fed by inflows from the Strėva River, and nine other rivulets.

**Strėva River** (Trakai and Kaunas district). Average current velocity is 0.1–0.3 m/s. The bottom is sandy mixed with silt. *Phragmites* sp. and *Nymphaea lutea* grow at the edges of the river. Water pH was 7.80 in the sampling site.

### Region 5

**Saterečius Pond** (Utena district). The pond is surrounded by marsh and mixed deciduous forest dominated by *Alnus* sp. In summer the pond is almost overgrown with macro-algae and such macrophytes as *Nymphaea lutea* and *Stratiotes aloides*. Water pH is 6.78.

**Žvirgždelis Lake** (Utena district) covers an area of 0.027 km²; the bottom is silt and *Phragmites* sp. dominates in littoral. Water pH is 7.03.

### Region 6

**Snaigynas Lake** (Lazdijai district). The lake covers an area of 2 km², with an average depth of 3 m. The shores are low and overgrown by shrubs and trees. The lake bottom is sandy in littoral. A small shallow stream flows out from this lake into Trikojis Lake.

### Sampling and observations

Statoblasts were taken by net from the surface of water and aquatic plants. Bryozoan colonies were collected from submerged branches, stones, and aquatic plants in the littoral of the water bodies. Statoblasts and bryozoan colonies were stored in 70% ethanol.

Identification of most species was based on morphology of statoblasts and colony (when colonies were available) using light and scanning electron microscopy (SEM) (Hitachi S-3400N).

Statoblasts characters included overall length and width, length and width of the fenestrae, and surface micro-sculpture of statoblasts. Abbreviations used for measurements are as follows:

**L/W** ratio of the statoblast,

**VfL** ventral fenestra length;

**VfW** ventral fenestra width;

**DfL** dorsal fenestra length;

**DfW** dorsal fenestra width.

Measurements were taken from SEM images with software Original Hitachi S-3400N Scanning Electron Microscope software ver 7.3.

Statoblasts were rinsed with distilled water several times then treated by KOH in order to remove any debris and cleaned using vortex for a few minutes. Statoblasts were prepared for scanning electron microscopy by simple drying without sputtering. The identification keys by Wood and Okamura (2005) were used.

### Material examined

The authors collected specimens during April through October 2015–2017. In total, 53 statoblasts and 8 colonies collected from 18 localities in Lithuania were examined. In addition, some data collected by Bronė Pajiedaitė (1932-1934) were included in this study for comparison. The representative specimens are deposited in the zoological collection of Biology Department of Vytautas Magnus University.

## Results

The survey of 18 water bodies yielded nine species of freshwater bryozoans (Table 1). Eight of these are classified with the Class Phylactolaemata: *Cristatella mucedo*, *Hyalinella punctata*, *Plumatella casmiana* Oka, 1907, *Plumatella fungosa*, *Plumatella fruticosa*, *Plumatella geimermassardi* Wood & Okamura, 2004 *Plumatella repens*, and *Plumatella emarginata*. The ninth species, *Paludicella articulata*, belongs to the Class Gymnolaemata.

### Taxonomy

### Class Phylactolaemata Allman, 1856

#### Order Plumatellida Allman, 1856

##### Family Plumatellidae Allman, 1856

###### Plumatella repens

Taxon classificationAnimaliaPlumatellidaPlumatellidae

(Linnaeus, 1758)

Fig. 2

####### Material examined.

Ten floatoblasts collected from ponds of Kaunas Botanical garden, Raudondvaris pond in April 2015, and Skyplaičiai lake, collected in June 2015; colonies collected from Raudondvaris and Rokai pond in June 2015 and July 2016 respectively. Sessoblasts were not found.

####### Description.

**Figure 2.**
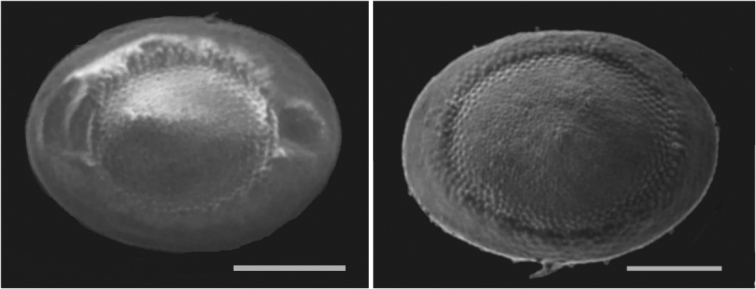
*Plumatella repens*. Scanning electron micrograph of floatoblasts showing dorsal valve (left) and ventral valve (right). Scale bars: 200 μm.

Colonies were about 5–8 cm size. The transparent branches of colonies were attached to the substratum for almost whole of their length. Floatoblasts were identified by the broadly oval shape and the absence of tubercles on the statoblast annulus (Fig. 2). Floatoblasts were 315–341 (325±3) µm long by 226–270 (252±4) µm wide; L/W ratio was 1.3; VfL 144–245 (187±14) µm; VfW 126–212 (168±10) µm(n=10); DfL 135–258 (178±14) µm; DfW126–212 (163± 9) µm (n=10). Fenestra of floatoblasts circular, covered with rounded tubercles that become less prominent towards the centre of fenestra. The annular nodules often described for this species have not yet been observed in Lithuanian material.

####### Distribution in Europe.

According to Økland and Økland (2005), Wood and Okamura (2005), *P. repens* is common in Britain, Ireland and Europe. Kaminski (1984) described *P. repens* as most common species in the studied lakes in Poland.

####### Remarks on habitat and ecology in Lithuania.

*Plumatella repens* has been the most commonly encountered species, with floatoblasts occurring in all surveyed sites, include lentic and stagnant habitats. Although colonies were found in only two ponds from listed sites, we have since become aware of colonies occurring in other lakes and ponds not listed here.

Pajiedaitė (1933) described *P. repens* as most common species in Lithuania, which can grow in various freshwater bodies. On the other hand, the exact locations of her collecting sites were not listed in her thesis. In addition, because of early difficulty in identifying this species, distribution reports prior to the mid-1980s are not necessarily reliable (Wood and Okamura 2005).

####### Remarks.

Lacourt (1968) postulated close relationship between *Plumatella repens* and *P. fungosa* based on a “short oval statoblasts” and molecular studies confirmed a close relationship between these species (Hirose et al. 2011). *Plumatella repens* can be confused with young colonies of *P. fungosa* (Wood and Okamura 2005). In addition, statoblasts of *P. repens* are similar to those of its congeners *P. nitens* Wood, 1996, *P. nodulosa* Wood, 2001, *P. orbisperma* (Kellicott, 1882), *P. recluse* Smith, 1992, and *P. rugosa* Wood, Wood, Geimer & Massard, 1998 (Massard and Geimer 2008a).

###### Plumatella geimermassardi

Taxon classificationAnimaliaPlumatellidaPlumatellidae

Wood & Okamura, 2004

Fig. 3

####### Material examined.

A few floatoblasts from Lampėdžiai Lake in April 2016. *P. geimermassardi* were recorded in Lithuania for the first time. However, the species is so far represented only by statoblasts.

####### Description.

**Figure 3.**
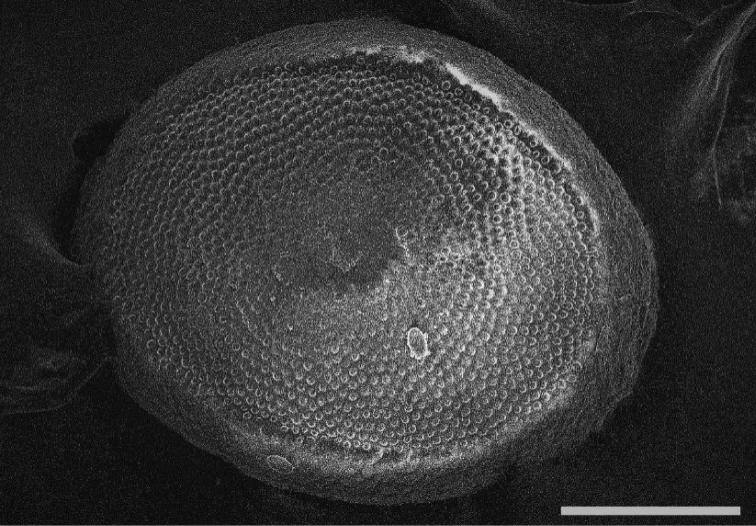
*Plumatella geimermassardi*. Scanning electron micrograph of the floatoblast ventral valve showing the uniformly narrow annulus. Scale bar: 100 μm.

Floatoblasts were identified by the large dorsal fenestra with tubercles and narrow annulus. The annulus at the poles is mostly as large as laterally and is covered by weakly visible tubercles (Fig. 3). Length and width of floatoblast were 311–325 (317±4) μm and 221–273 (244±15) μm (n=3) respectively. L/W ratio 1.3; DfL 199–205 (202±3) μm; DfW 174–201 (187±13) μm (n=3); VfL 200–254 (227±26) μm and VfW 185–198 (192±6) μm (n=3).

####### Distribution in Europe.

*Plumatella geimermassardi* is known from England, Ireland, Belgium, southern Norway, northern Germany, Italy and Finland (Wood and Okamura 2005).

####### Remarks.

Floatoblasts of *P. geimermassardi* are among the smallest floatoblasts among all European plumatellids with an average length of around 320 µm (Wood and Okamura 2004). The uniformly narrow annulus offers an easy identifying feature characteristic for broad floatoblasts in this species. The relatively large area of dorsal and ventral fenestrae is matched only by those of *P. nitens* or *Stephanella hina* on other continents (Wood 1996; Toriumi 1955).

###### Plumatella fungosa

Taxon classificationAnimaliaPlumatellidaPlumatellidae

(Pallas, 1768)

Fig. 4

####### Material examined.

**Figure 4.**
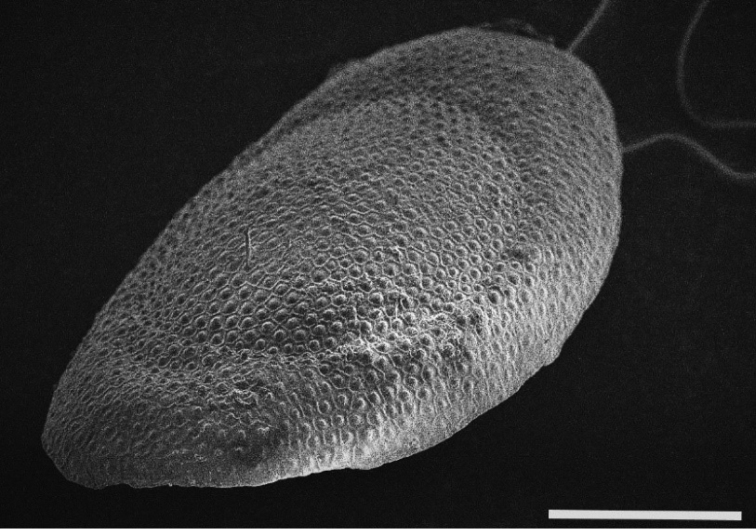
*Plumatella fungosa*. Scanning electron micrograph showing characteristic tubercles on the floatoblast annulus. Scale bar: 100 μm.

A floatoblasts collected from Linksmakalnis pond (June 2015) and Maišia stream (April 2015). Colony from Aristava pond (locates in Kėdainiai district 55°17'07.1"N, 24°04'28.6"E and it is not included in general list of studied sites during this survey) was taken in June 2017 (Fig. 4).

####### Description.

The colony dark, spindle shaped, and large (15–17 cm), formed on stems of reeds (*Phragmites*). Examined floatoblasts exhibited characteristic tubercles on the floatoblast annulus (Fig. 4) and a ridge-like suture between the dorsal and ventral valves. Dorsal floatoblast tubercles were larger on the fenestra than on the annulus. The length of floatoblasts was 324–368 (339±5) µm; width 220–290 (254±8) µm (n=8), L/W ratio 1.3; DfL 130–160 (147±4) µm (n=6); DfW 125–161 (144±5) µm (n=6); VfL 214–250 (227±4) µm and VfW 205–228 (214±2) µm (n=6). However, dimensions of *P. fungosa* floatoblasts provided by Pajiedaitė (1933), were slightly larger: 470 μm × 290 μm. Pajiedaitė also recorded the variability in sessoblast dimensions from different localities: 790 μm × 470 μm in Nevėžis river (Kaunas district); 480 μm × 370 μm in Lake Aukštadvaris (Trakai district), and 580 μm × 420 μm in Snaigynas Lake (Lazdijai district) (Pajiedaite 1933). During current study sessoblasts were not found.

####### Distribution in Europe.

According to Wood and Okamura (2005) *P. fungosa* is widespread in Europe. It has been recorded from several places in southern Sweden and Finland; it is common in Denmark and has been reported from Iceland (Økland and Økland 2005) and Poland (Kaminski 1984).

####### Remarks on habitat and ecology in Lithuania.

During this survey, floatoblasts of *P. fungosa* were found in seven water bodies from 18 surveyed, with prevalence in stagnant water, with neutral to slightly alkaline pH 7.01–8.15 (Table 1). Pajiedaitė (1933) described the colonies in Kaunas Lagoon, Nevėžis River (Kaunas district) and Dubysa River (Šiauliai district). She noted that *P. fungosa* often occurred in polluted water and described colonies, found in old port of Kaunas city, where water was polluted by oil of ships and trash. A similar observation has been made by other authors (Bushnell 1966; Geimer and Massard 1986). Based on the available data, we consider *P. fungosa* to be prevalent in Lithuania.

####### Remarks.

Large bulky colonies of *P. fungosa* are easily recognizable freshwater bryozoan species in Europe (Wood and Okamura 2004). Floatoblasts of *P. fungosa* are lateralyasymmetrical and distinctfrom the symmetrical floatoblasts of *P. repens* and *P. rugosa*. Molecular studies showed a close relationship between *P. repens* and *P. fungosa* (Hirose et al. 2011).

###### Plumatella emarginata

Taxon classificationAnimaliaPlumatellidaPlumatellidae

(Allman, 1844)

Fig. 5

####### Material examined.

A few floatoblasts from Šventupė pond were collected in July 2016.

####### Description.

Floatoblasts elongated in shape, with a circular ventral fenestra and small dorsal fenestra, covered by tubercles. Floatoblasts were 357–489 (407±18) µm long and 197–235 (216±6) µm (n=6) wide, L/W ratio 1.9; DfL 97–125 (107±9) µm; DfW 60–82 (68±7) µm (n=3); VfL 101–184 (149±9) µm and VfW 110–162 (133±4) µm (n=6). The approximate size of statoblasts provided by Pajiedaite (1933) was 560 µm long and 260 µm wide.

**Figure 5.**
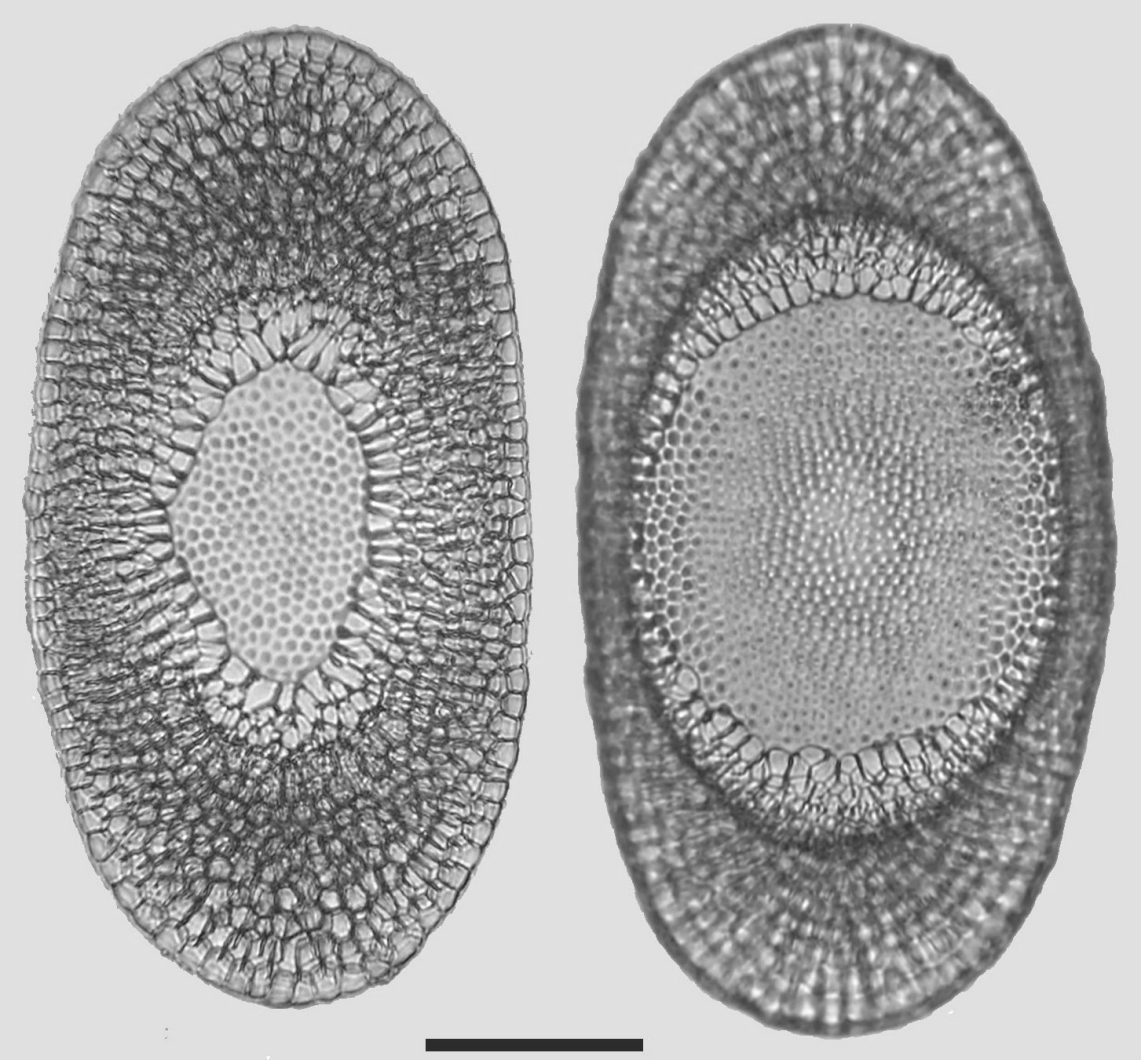
*Plumatella emarginata* floatoblast valves. The dorsal valve (left) has a characteristically small central fenestra; in the ventral valve (right) the fenestra is nearly circular. Scale bar: 100 µm.

####### Distribution in Europe.

Geimer and Massard (1986) defined the range of this species to include most of Europe. Økland and Økland (2005) considered *P. emarginata* to be a southern species, with limited distribution in Norway and Sweden.

####### Remarks on habitat and ecology in Lithuania.

Pajiedaitė (1933) described morphology of *P. emarginata* colonies, but her text is not clear about the location of collection sites. However, the statoblasts she found were recorded from Paštys Lake (55°42'36"N, 25°41'48"E), Satarečius pond and Dubysa River (Kaunas district, 55°12'12"N, 23°30'28"E).

In our survey only a few statoblasts were found in Šventupės pond (Table 1). Wood and Okamura (2005) noted that *P. emarginata* is particularly tolerant of rapidly-flowing water. The occurrence of floatoblasts in the Neries River (Kaunas district - not included in this study) is consistent with this observation, although colonies were not found. From our data *P. emarginata* would be considered uncommon in Lithuania, although this should be verified through further surveys.

####### Remarks.

The species is widely distributed throughout the Holarctic (Wood and Okamura 2005), although some reports may have confused it with similar species, *P. mukaii* or *P. reticulata* (Massard and Geimer 2008a).

###### Plumatella casmiana

Taxon classificationAnimaliaPlumatellidaPlumatellidae

(Oka, 1908)

Fig. 6A, B

####### Material examined.

Floatoblasts, leptoblasts, and colony from Linksmakalnis pond collected from submerged branches in 20 July 2016.

####### Description.

**Figure 6.**
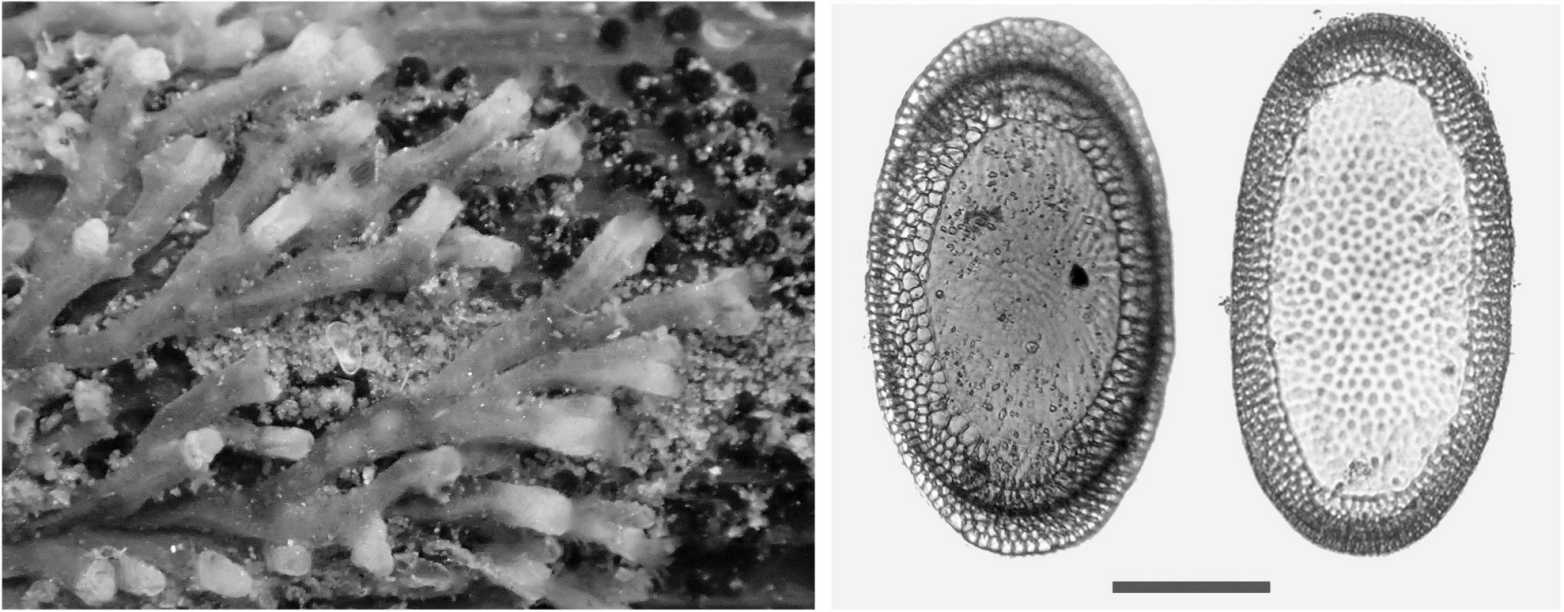
*Plumatella casmiana*. **A** Portion of a colony showing crowded zooids almost entirely attached to the substratum **B** Dorsal valve of floatoblast (left) and leptoblast (right). Scale bars: 1 mm (**A**), 100 µm (**B**).

Colony was about 5–6 cm long. Branches of colony are short, almost entirely attached to the substrate. The terminal parts of branches are semi-transparent and whitish. The floatoblasts were recognized by the distinctly elongated shape of the fenestra on both valves. Both capsuled floatoblasts and the distinctive leptoblasts were found, along with associated colonies (Fig. 6A, B). The surface fenestra of capsulated floatoblasts was almost smooth. Length of floatoblasts 345–432 (397±15) µm; width 188–260 (214±14) µm), L/W ratio 1.8; DfL 112–198 (154±15) µm; DfW 90–135 (113±7) µm; VfL 174–236 (205±12) µm; VfW 150–195 (167±8) (n=5). Leptoblasts (Fig. 6B, right side) have a uniformly narrow annulus and extensive oval fenestrae; which length was at least 1.5 times its width.

####### Distribution in Europe.

*Plumatella casmiana* is currently known throughout most of Europe (Massard and Geimer 1995b).

####### Remarks on habitat and ecology in Lithuania.

This is the first reported occurrence of *P. casmiana* in Lithuania. Floatoblasts of *P. casmiana* were recorded in almost half of the investigated water bodies (Table 1). However, colonies were found in Linksmakalnis Pond only.

####### Remarks.

Beyond Europe *P. casmiana* is widely distributed through Asia, North America, Africa, and very likely other continents as well (Wood and Okamura 2005). A unique feature is the appearance of floatoblasts lacking the inner capsule (Figure 6B, right side). This so-called leptoblast is capable of hatching immediately after release from the colony, enabling populations to grow very rapidly each season. Colonies also produce conventional capsuled floatoblasts (Figure 6B, left side) which retain the obligatory dormancy period.

###### Plumatella fruticosa

Taxon classificationAnimaliaPlumatellidaPlumatellidae

(Allman, 1844)

Fig. 7A, B

####### Material examined.

Colony from Rokai pond (Kaunas district) found in June 2016; floatoblasts from pond of Kaunas Botanical garden collected in July and August 2016.

####### Description.

The colony measured approximately 3 x 4 cm and had sparse, narrow and upright branches. Free statoblasts are long and narrow, exhibiting a length at least twice the width: 432–496 (459±8) μm long and 187–220 (203±4) μm (n=10) wide; L/W ratio 2.2; DfL 120–320 (197±19) (n=10) μm; DfW 56–100 (75±6) (n=6) μm; VfL 211–313 (266±21) (n=4) μm and VfW 74–128 (108±17) μm (n=3). Sessoblasts were not found during this study. According to Pajiedaitė (1933) the average size of the floatoblasts was 590 μm long and 230 μm wide.

####### Distribution in Europe.

*Plumatella fruticosa* is considered to be widespread, especially in northern portion of Europe (Økland and Økland 2005). It is considered common in Poland (Kaminski 1984).

####### Remarks on habitat and ecology on Lithuania.

**Figure 7.**
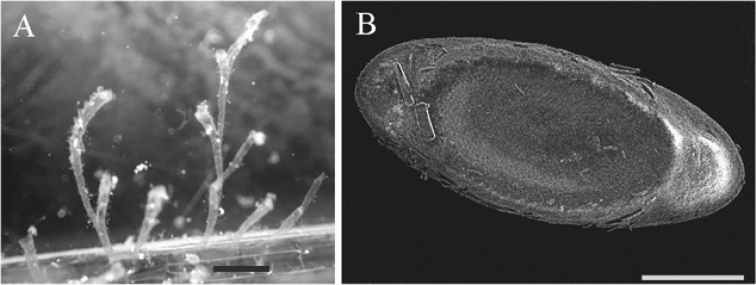
*Plumatella fruticosa*. **A** Colony showing upright zooids and branches (**B**) Scanning electron micrograph showing floatoblast ventral valve with characteristic long, narrow shape. Scale bars: 3 mm (**A**); 200 μm (**B**).

Pajiedaitė collected colonies in Dubysa river (Šiauliai district) and Satarečius pond (Utena district) (Pajiedaitė 1933) At first glance we could state that *P. fruticosa* is common in Lithuania, since during this survey statoblasts were found in most water bodies. However, we found colonies only in Rokai pond with sandy-mud bottom and stones in the littoral (Table 1, Fig. 7). Thus, it is possible, that statoblasts are spread by waterfowl among various ponds and lakes, but these may not be the preferred environment for growing colonies (Økland and Økland 2005).

####### Remarks.

The combined statoblast characteristics (large length/width ratio, strong asymmetry of floatoblast and sessoblast, narrow fenestra on dorsal floatoblast valve) distinguish *P. fruticosa* from all other plumatellid species (Ricciardi and Reiswig 1994). Molecular results provided by Hartikainen (Hartikainen et al. 2013) imply that *P. fruticosa* is not a plumatellid and provide evidence for polyphyly in *Plumatella*. However, the position of *P. fruticosa* remains unresolved (Hartikainen et al. 2013).

###### Hyalinella punctata

Taxon classificationAnimaliaPlumatellidaPlumatellidae

(Hancock, 1850)

Fig. 8

####### Material examined.

A few statoblasts from Veršvio stream were found in August 2015 Unfortunately, these were later lost before critical dimensions could be taken.

####### Description.

Colonies were not observed, and species was identified according floatoblasts. The statoblasts are larger than any other plumatellid species and show crowded tubercles on the fenestrae of both valves. Pajiedaitė (1933) described floatoblasts by oval shape, with length 440 µm and width 230 µm. These dimensions were slightly smaller than 500 µm and 350 µm suggested by Wood and Okamura (2005). Length and width of measured statoblasts during current study was 425–459 (444±7) and 280–299 (290±4) µm, respectively (n=5).

**Figure 8.**
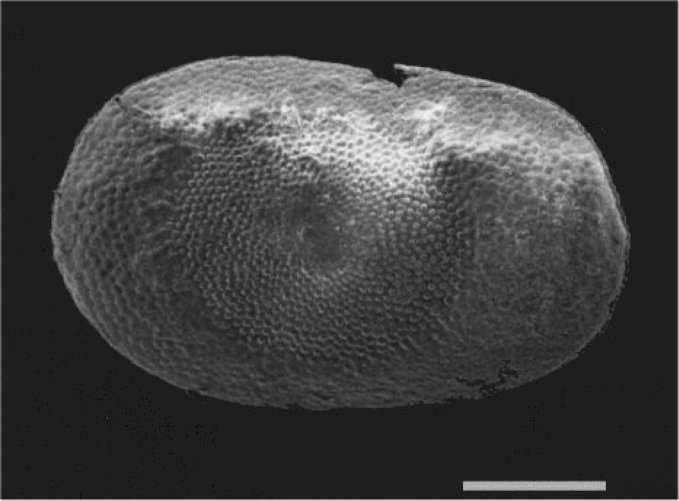
*Hyalinella punctata*. Scanning electron micrograph showing the floatoblast ventral valve. Scale bar: 300 μm.

####### Distribution in Europe.

*Hyalinella punctata* has been widely reported worldwide, including neighbouring Poland (Kaminski 1984), but verified specimens are known only from Britain, Ireland, Europe, North America and northern Asia (Wood and Okamura 2005).

####### Remarks on habitat and ecology in Lithuania.

Few small colonies of *H. punctata* were described on *Nymphaea lutea* leaves in small lakes in the Zarasai district (55°44'50"N, 25°50'4"E) and Dubysa river (Šiauliai district; 55°51'29"N, 23°08'31"E) by Pajiedaitė (1933). During the present survey, floatoblasts of *H. punctata* were recorded only in the Veršvio stream (Table 1). The available data are not sufficient to estimate the prevalence and frequency of this species in Lithuania.

####### Remarks.

Hancock (1850) described colonies of *H. punctata* as “thick and transparent with less profuse branching than in *Plumatella* and produce only floatoblasts, while individual zooids are indistinct, usually arranged linearly and lack interzooidal septa”. In fact, features distinguishing *Hyalinella* from *Plumatella* are not clear-cut (Hirose and Mawatari 2011), because the diagnosis of *Hyalinella* is based on the transparency and thickness of the colony wall (ectocyst), but the condition of the ectocyst depends to some extent on environmental factors (Wood and Okamura 2005, Hirose and Mawatari 2011). Generic placement of some species between *Plumatella* and *Hyalinella* has remained unstable (Hirose and Mawatari 2011).

##### Family Cristatellidae Allman, 1856

###### Cristatella mucedo

Taxon classificationAnimaliaPlumatellidaCristatellidae

(Cuvier, 1798)

Fig. 9

####### Material examined.

Colony from Snaigynas lake (Lazdijai district) collected in July 2016, floatoblasts from Rokai pond found in September 2016.

####### Description.

**Figure 9.**
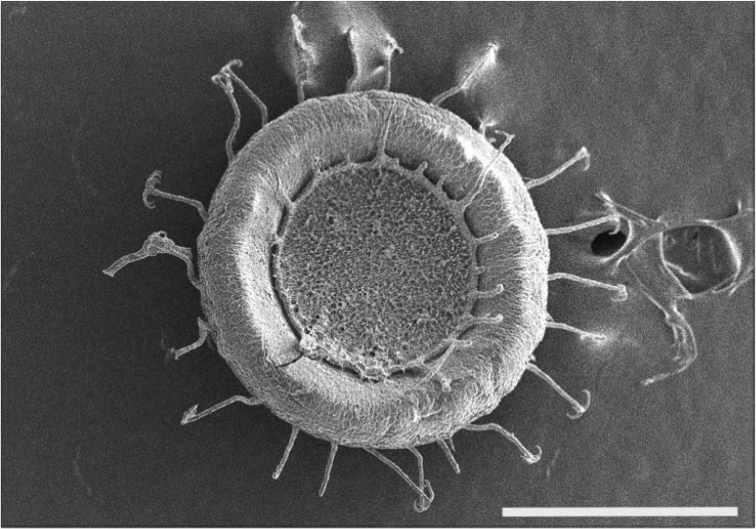
*Cristatella mucedo*. Scanning electron micrograph showing floatoblast with characteristic spines. Scale bar: 500 μm.

Colonies of *C. mucedo* are recognized by their elongated shape and colourless, transparent body wall. The length of colonies found varied from 5 to 10 cm (Pajiedaitė 1933; this study). The large statoblasts are easily recognized by circular form with hooked spines radiating from the edges of the fenestrae on both valves (Fig. 9). Diameter of statoblasts was about 1 mm.

####### Distribution in Europe.

*Cristatella mucedo* is a common species in Lithuania, with a Holarctic distribution, occurring in Britain, Ireland, Europe, Asia and North America (Økland and Økland 2000; Wood and Okamura 2005).

####### Remarks on habitat and ecology in Lithuania.

During this survey a few colonies of *C. mucedo* occurred in South Lithuania (Snaigynas lake), but statoblasts were found in various water bodies of different regions of the country (Table 1). Pajiedaitė (1933) noted that *C. mucedo* more often occurred in South Lithuania. She found colonies of *C. mucedo* without statoblasts in June/July and noted that statoblasts inside colonies appeared in first part of August. Numerous colonies with statoblasts were found at the end of September and they died late autumn once the water temperature dropped to 3 °C in November 1932 (Pajiedaitė 1933).

####### Remarks.

A more detailed discussion of the ecology and life history of *C. mucedo* can be found in Okamura (1997).

### Class Gymnolaemata Allman, 1856

#### Order Ctenostemata Busk, 1852

##### Family Paludicellidae Allman, 1844

###### Paludicella articulata

Taxon classificationAnimaliaCtenostemataPaludicellidae

(Ehrenberg, 1831)

Fig. 10

####### Material examined.

Two colonies from the outlet of Snaigynas Lake (Lazdijai district) were found in May 2017. Hibernaculae were not found.

####### Description.

The species was recognized by the slender colony branches forking at wide angles and often growing free from the substratum. Colonies were small, about 2–3 cm. Branches of colony were transparent and shiny. Zooids 1.0–1.5 mm in length with 16 tentacles on a circular lophophore were described by Pajiedaitė (1933).

**Figure 10.**
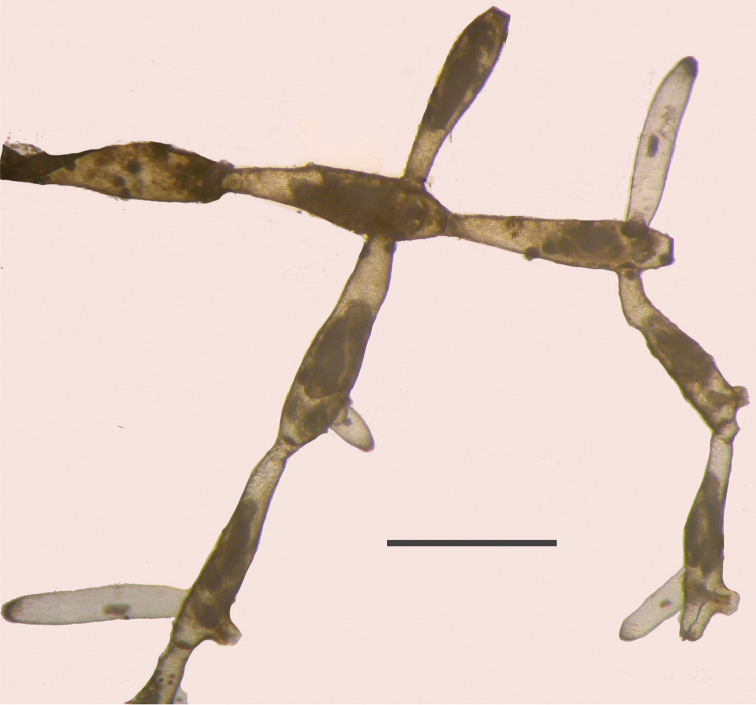
Fragment colony of *Paludicella articulata*. Scale bar: 1 mm.

####### Distribution in Europe.

*Paludicella articulata* is known worldwide (Wood and Okamura 2005). However, the species has not been found in Poland (Kaminski 1984).

####### Remarks on habitat and ecology in Lithuania.

*Paludicella articulata* was recorded by Pajiedaite (1933) in only two localities: Paštys Lake (Utena district) (55°42'36"N, 25°41'48"E) and Satarečius pond (Utena district). Since *P. articulata* tolerates cold temperatures (Økland and Økland 2005) and prefers flowing water (Wood and Okamura 2005) it was surprising finding of this species in stagnant Satarečius pond together with *C. mucedo*. Coexistence of the two species was also noted by Pajiedaitė (1933), who explained it by different local conditions in the same pond; colonies of *P. articulate* were observed only near a small stream flowing into the pond. Otherwise, she noted that C. *mucedo* was mostly observed in the warmer waters of Central and South Lithuania.

Økland and Økland (2005) showed positive co-occurrence of these two species in Norway.

During this survey *P. articulata* was found in the outlet of Snaigynas lake, which is of glacial origin and characterised by low temperature.

####### Remarks.

Colonies of *P. articulata* consist of sometimes creeping but more often elongated, mostly erect, slender zooids. There are normally three adjacent zooids: one distal and two lateral ones (Davenport 1891) The contiguous arrangement of the zooids and the subterminal 4-sided zooecial orifice readily distinguish the species from its closest relative, *Pottsiella erecta* (Ricciardi and Reiswig 1994).

## Discussion

Overall nine species of freshwater bryozoans are now known from Lithuania. This contrasts with about 19 species reported from Europe and about 13 species from the Baltic area (MassardGeimer and MassardGeimer 2004; Nikulina 2006). Given the fact that only a relatively few water bodies of Lithuania have been investigated so far it is likely that the final tally of species will be higher.

The majority of surveyed pools were stagnant, neutral or slightly alkaline (Table 1) and should have been suitable for bryozoans to grow successfully. However, while intact colonies were found only in few sites, statoblasts were widely distributed. The rarity of colonies in water pools might be explained by fluctuating climatic conditions, especially the alternation of drought and rainfall. The similar process under Lithuanian conditions was described by Pajiedaitė (1933), who found *P. articulata* and *P. repens* colonies in Paštys Lake in 1931, but completely absent the following year. Pajiedaitė (1933) suggested this disappearance may have been due to rising of water level after rainfall in Paštys Lake. She wrote that bryozoans are sensitive “creatures” and cannot survive such drastic environmental change. She went on to describe a similar situation in Nevėžis River in 1932, where after week of rainfall, nearly all bryozoan colonies had died (Pajiedaitė 1933). Jong-Yun-Choi et al. (2015) documented the negative effects of heavy rainfall in Korea on colonies of *Pectinatella magnifica* (Leidy, 1851).

Another possible reason of finding small number of colonies could be the lack of suitable substratum for the attachment of colonies. Because bryozoans are sessile organisms, they absolutely require a solid, inert substratum on which to grow (Ryland 1970). For example, we have found statoblasts of five bryozoan species in a pond at the Kaunas botanical garden (Table 1). However, colonies have never been observed there, possibly due to lack of solid substratum to which bryozoan colonies can attach. The presence of statoblasts could be the result of waterfowl, which are known to transport them from one site to another (Wood and Okamura 2005).

With this study, we have now recorded 13 species of freshwater bryozoans recorded in Baltic area: *C. mucedo*, *P. magnifica*, *Fredericella indica* Annandale, 1909, *Fredericella sultana* (Blumenbach, 1779), *Lophopus crystallinus* (Pallas, 1768), *H. punctata*, *P. casmiana*, *P. emarginata*, *P. fruticosa*, *P. fungosa*, *P. geimermassardi*, *P. repens*, *Stolella indica* Annandale, 1909, and *P. articulata* (MassardGeimer and MassardGeimer 2004; Nikulina 2006)

All bryozoan species documented in Lithuania are common and widely distributed through Europe. The composition of species found through this survey was similar to that recorded 86 years ago, with the exception of finding two additional species for Lithuania. The status of *P. repens* and *P. fungosa* as common freshwater bryozoan species, and *P. articulata* as rare, have not changed for almost a century.

Curiously, this survey did not encounter *F. sultana*, which is otherwise well known in northern Europe, Britain and Ireland (Geimer and Massard 1986; Wood and Okamura 2005). Also absent was the large gelatinous species, *P. magnifica*, which is currently expanding its range across Europe and is already reported from areas including Hungary (Szekeres et al. 2013), Germany (Grabow 2005), Czech Republic (Rodriguez and Vergon 2002; Balounova et al. 2011), and Poland (Balounová et al. 2011). Additional bryozoan species that might be expected in Lithuania include *L. crystallinus*, which is recorded in neighbouring countries as Belarus, Kaliningrad and Poland (Nikulina 2006); also, *P. rugosa*, *P. reticulata* Wood, 1988, and *F. indica*. Since the brackish species, *Victorella pavida* Saville Kent, 1870, is known in neighbouring Latvia, it is likely to be found also in Lithuania.

In summary, we believe that the list of freshwater bryozoa presented here is not final. Lithuania is an extremely watery region; there are more than 3000 lakes of a wide variety of sizes and many rivers flow across the country. Therefore, it is very likely, that further research will reveal additional species.

## Supplementary Material

XML Treatment for Plumatella repens

XML Treatment for Plumatella geimermassardi

XML Treatment for Plumatella fungosa

XML Treatment for Plumatella emarginata

XML Treatment for Plumatella casmiana

XML Treatment for Plumatella fruticosa

XML Treatment for Hyalinella punctata

XML Treatment for Cristatella mucedo

XML Treatment for Paludicella articulata
